# Optimized SPE–UPLC–MS/MS Method for Sensitive Determination of Cereulide in Complex InfantFormula Matrices

**DOI:** 10.3390/toxins18050222

**Published:** 2026-05-08

**Authors:** Zixiao Zhou, Ziyi Wang, Chundi Mu, Yan Qi, Jing Zhang, Xia Cui, Sai Fan, Jing Xiao, Rong Zhao

**Affiliations:** 1 Department of Physics and Chemistry, Beijing Center for Disease Prevention and Control, Beijing 100013, China; 2 College of International Education, Beijing University of Agriculture, Beijing 102206, China; 3 China National Center for Food Safety Risk Assessment, Beijing 100022, China

**Keywords:** cereulide, infant formula, SPE, UPLC-MS/MS

## Abstract

Cereulide is a heat-stable cyclic depsipeptide toxin produced by *Bacillus cereus* and is responsible for foodborne emetic syndrome. Recent reports of *Bacillus cereus* contamination and cereulide occurrence in infant formula have raised increasing food safety concerns. Due to the immature immune and metabolic systems of infants, exposure to cereulide through contaminated formula may lead to potential health risks. However, direct application of existing cereulide analytical methods to infant formula remains challenging because of the unique processing technologies, encapsulated nutrients, and variable matrix composition of this product category, which may hinder toxin release and cause significant matrix interference. In practical analysis, inter-laboratory comparisons revealed that existing methods exhibited relatively large deviations and insufficient sensitivity, making them not specifically optimized for infant formula matrices. The present study was motivated by the need for a matrix-specific, sensitive, and reliable analytical method for cereulide determination in infant formula. In this study, a method based on solid-phase extraction coupled with ultra-performance liquid chromatography–tandem mass spectrometry (SPE–UPLC–MS/MS) was developed and validated. To improve the applicability of cereulide analysis to infant formula, this method incorporates a hydration-assisted extraction step tailored to infant formula, which increased the detected cereulide response by approximately fourfold, together with optimized SPE clean-up and improved chromatographic conditions to reduce matrix effects and enhance quantitative reproducibility. The method showed good linearity (0.1–10 μg·L^−1^, R^2^ > 0.999), low values for limit of detection (LOD) (0.03 μg·kg^−1^) and limit of quantification (LOQ) (0.1 μg·kg^−1^), and acceptable recoveries (94.4–110.3%) with RSDs below 3.7%. The developed method was successfully applied to commercial infant formula samples, and cereulide-positive samples were identified. This method provides a reliable analytical tool for the monitoring of cereulide in infant formula and contributes to improved food safety surveillance and exposure risk assessment.

## 1. Introduction

*Bacillus cereus* is a Gram-positive bacterium widely distributed in natural environments, where it is found in soil, sediments, aquatic habitats, on plant surfaces, etc. Therefore, it has frequently been detected in meat products, seafood, dairy products, and various ready-to-eat foods [1,2,3]. This widespread bacterium can induce two types of food-associated gastrointestinal diseases: (1) a diarrheal syndrome caused by relatively heat-labile enterotoxins; (2) an emetic and nausea syndrome caused by the highly heat-stable cereulide [4,5,6].

Recently, contamination of infant formula products by *Bacillus cereus* has attracted increasing global attention. In 2025, several international recalls of powdered infant formula were reported due to contamination associated with *B. cereus* and its emetic toxin cereulide, affecting multiple commercial products distributed in Asia, Europe and other regions. Investigations suggested that the contamination was related to raw ingredients used during manufacturing. Considering the high thermal stability of cereulide, it cannot be inactivated during conventional formula preparation using hot water, which increases the potential exposure risk for infants. Clinical reports indicate that ingestion of cereulide-contaminated foods can cause rapid-onset vomiting and gastrointestinal distress, and severe symptoms may occur particularly in infants younger than six months [3,7]. These incidents highlight the importance of reliable analytical methods for the detection of cereulide in infant formula to support food safety surveillance and risk management.

**Figure 1:**
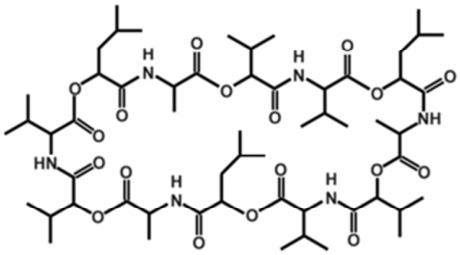
Structure of the depsipeptide toxin cereulide, the causative agent for the emetic type of *B. cereus* food-borne intoxications.

Cereulide, produced by *Bacillus cereus*, is a cyclic dodecadepsipeptide composed of three repeating tetradepsipeptide units (D-O-leucine–D-alanine–L-O-valine–L-valine) (as shown in Figure 1) [8,9]. Due to its cyclic structure, it is chemically stable and exhibits pronounced thermal stabilityand acid resistance (stable from pH 2 to 11) , being able to withstand heating at 121 °C for 90 min as well as degradation by digestive enzymes and acidic conditions [10]. Accordingly, it is difficult to eliminate from foods and remains resistant to detoxification in the animal gastrointestinal tract after cereulide entering the food chain [10,11]. Documented toxicological properties of cereulide include inhibition of insulin secretion, damage to pancreatic β-cells and hepatocytes, and disruption of intestinal epithelial integrity [12,13]. Chronic exposure (oral administration of 50/200 μg·kg^−1^ in mice for 28 days) has been shown to induce oxidative stress and inflammation of the liver, and may act synergistically with other toxins to further aggravate intestinal injury [14,15].

Various analytical approaches have been developed for the detection of cereulide. Biological assays enable detection of total toxin activity and some allow semi-quantitative analysis. However, these assays cannot identify the specific toxin responsible for gastrointestinal illness, and are prone to interference from similar molecules. Furthermore, partial complex procedures are involved that are difficult to perform in general laboratories [16]. Molecular methods such as PCR allow rapid detection of the *ces* gene in *Bacillus cereus*, indirectly providing the toxigenic potential of a strain. Nevertheless, PCR can only confirm the presence of toxin-producing genes and cannot evaluate the actual expression level or concentration, limiting its applicability as evidence in foodborne intoxication. In contrast, chemical analytical methods, particularly LC–MS or UPLC–MS/MS, are regarded as the gold standard for the detection and quantification of cereulide. Emerging approaches have also been explored, such as matrix-assisted laser desorption/ionization time-of-flight mass spectrometry (MALDI-TOF MS) [17]. Internationally, ISO 18465:2017 has been established and implemented, recommending stable isotope dilution analysis using ^13^C_6_-cereulide as an internal standard for accurate quantification [18,19]. Currently, no mandatory national standard for cereulide determination is available in China, although the group standard T/WSJD 57-2024 and related research methods have been issued.

Although ISO provides an established analytical framework for cereulide determination, infant formula presents matrix-specific analytical challenges due to its complex composition and processing characteristics. In actual application, the same cereulide-positive sample was analyzed by different laboratories using the existing method, and the results showed substantial inter-laboratory variability, with notably high RSD. This indicates that methods developed for conventional matrices may have limitations when applied to infant formula samples; this observation may be attributed to the complexity of the infant formula matrix, suggesting that further optimization could improve method performance for this specific application. Infant formula is produced through complex industrial processes and typically contains proteins, lipids, carbohydrates, and a wide range of fortified nutrients [20,21]. In many formulations, functional ingredients and micronutrients are incorporated using encapsulation or protein-based embedding technologies to improve stability and bioavailability [20]. Such structural characteristics may hinder the efficient release of cereulide during extraction, thereby reducing extraction efficiency and affecting quantitative accuracy [22,23]. In addition, modern infant formulas often contain numerous added nutritional components, including vitamins, minerals and bioactive compounds, which substantially increase matrix complexity. These co-extracted substances can generate strong matrix interferences during mass spectrometric analysis, posing significant challenges for sample purification and reliable quantification. Furthermore, cereulide is a relatively large and hydrophobic cyclic depsipeptide, whose chromatographic behavior differs from that of many common food contaminants. Inadequate chromatographic conditions may result in poor peak shape or retention behavior, which can compromise analytical sensitivity and reproducibility.

Therefore, analytical methods developed for conventional food matrices may not be directly suitable for infant formula. The key analytical gap lies in the lack of a validated, matrix-specific method capable of efficiently releasing cereulide from processed and encapsulated formula matrices while simultaneously minimizing matrix effects during UPLC–MS/MS analysis. Motivated by this need, the present study aimed to develop and validate a sensitive SPE–UPLC–MS/MS method specifically for infant formula. The novelty of this work centers on the systematic optimization of hydration-assisted extraction, SPE loading and washing conditions, and chromatographic separation for this complex matrix, improving its applicability to infant formula.

## 2. Results and Discussion

Infant formula, as a relatively complex matrix, contains proteins, fats, and carbohydrates, such as whey proteins, vegetable oils, and lactose, requiring detailed optimization of sample pretreatment to minimize matrix effects that may lead to low sensitivity and inaccurate results. In addition, infant formula is supplemented with a wide range of fortified nutrients, including various vitamins, DHA/ARA, minerals, etc. The diversity of these components introduces multi-dimensional matrix interferences, which may further affect analytical reliability if not properly controlled. Moreover, encapsulation technologies are commonly applied during processing, in which nutrients are embedded within whey protein structures, posing additional challenges for the efficient extraction of target analytes. Therefore, particular attention must be paid to optimizing the extraction stage. Based on these considerations, the effects of different factors on the extraction and purification methods were systematically investigated, and a suitable sample preparation protocol for the determination of cereulide in infant formula was established.

### 2.1. Optimization of the Extraction Method

According to typical manufacturer instructions, infant formula is usually reconstituted at a powder-to-water ratio of approximately 1:7–1:8. For a 2 g infant formula powder sample, this ratio would require approximately 14–16 mL of water for reconstitution before organic solvent extraction, leading to increased consumption of organic solvents, higher loading on the SPE cartridges, longer concentration times, and a greater risk of analyte loss during transfer steps. These factors significantly reduce the economic efficiency and operational convenience of the method. Therefore, this study attempted to reduce the extraction volume by appropriately increasing the powder to water ratio.

Preliminary experiments showed that when the powder-to-water ratio was ≥1:1.5, the hydration of the milk powder was insufficient. Under limited water conditions, casein micelles did not fully swell and formed poorly soluble aggregates that adhered to the container walls (wall-adhesion phenomenon), resulting in a non-uniform dispersion system. These inadequately hydrated particles may entrap cereulide and decrease extraction efficiency, while residual particles may also interfere with subsequent centrifugation. Based on these observations, the optimization range was set at powder-to-water ratios from 1:2 to 1:4, balancing sufficient hydration and practical operability.

In this study, 2 g cereulide-positive infant formula sample was added with 1 ng·mL^−1 13^C_6_-cereulide standard solution. Deionized water (45 °C) was added at powder-to-water ratios of 1:2, 1:2.5, 1:3, and 1:4, respectively. The mixtures were vortexed for 5 min for complete hydration, and then mixed with acetonitrile for protein precipitation. After centrifugation, the extracts were processed according to the ISO 18465:2017. Each experimental condition was performed in triplicate (n = 3).

**Figure 2:**
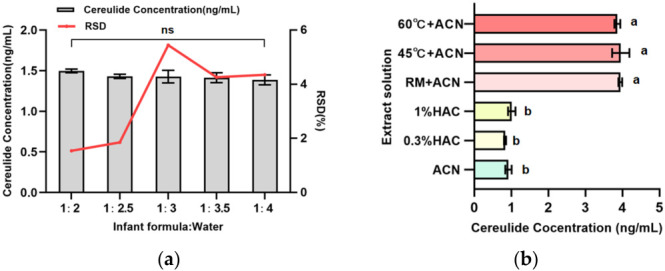
Effects of extraction conditions on cereulide extraction efficiency in infant formula. (**a**) Cereulide concentrations and RSDs obtained under different powder-to-water ratios. A 2 g infant formula sample was hydrated with deionized water at powder-to-water ratios of 1:2, 1:2.5, 1:3, 1:3.5, and 1:4, followed by acetonitrile extraction. ns indicates no significant difference among infant formula-to-water ratios by one-way ANOVA (*p* > 0.05). (**b**) Cereulide concentrations obtained using different extraction conditions, including direct acetonitrile extraction, acidified acetonitrile extraction, and hydration-assisted extraction at different temperatures before acetonitrile addition. Data are presented as mean ± SD (n = 3). Different lowercase letters (a and b) indicate the results of multiple comparisons after one-way ANOVA; groups labeled with different letters are significantly different (*p* < 0.05), whereas the same letter indicates no significant difference.

The cereulide concentration in positive samples, quantified using the internal standard method, ranged from 1.33 to 1.51 ng·mL^−1^ under different powder-to-water ratios (Figure 2a). The mean measured concentrations were 1.50, 1.43, 1.40, 1.41, and 1.39 ng·mL^−1^ for the 1:2, 1:2.5, 1:3, 1:3.5, and 1:4 groups, respectively. One-way ANOVA showed no significant differences among the groups (*p* > 0.05), suggesting that the powder-to-water ratio had limited influence on cereulide extraction efficiency within the range of 1:2–1:4.

Please change to the following: Precision evaluation indicated that the RSDs for the 1:2 and 1:2.5 groups were 1.54% and 1.85%, respectively, which were lower than those for the 1:3 group (5.44%), 1:3.5 (4.26%) and the 1:4 group (4.35%), and most values were close to or below the 5% threshold commonly accepted in analytical chemistry. These results suggest that a moderately higher milk powder proportison can reduce the influence of aqueous phase volume variation on dispersion homogeneity, decreasing variability among replicates. In contrast, excessive water addition dilutes the milk powder system, destabilizes the colloidal structure, and diminishes the reproducibility of protein precipitation, resulting in decreased precision. Because no statistically significant difference in extraction efficiency was observed between the 1:2 and 1:2.5 ratios, both conditions were considered comparable in terms of analytical response. The 1:2 ratio was selected for subsequent experiments because it required a smaller extraction volume, reduced the SPE cartridge loading volume, shortened the concentration process, and maintained satisfactory precision. For the 1:2 ratio group, the total extraction volume was 8 mL (4 mL water + 4 mL acetonitrile), about 20% less than that of the 1:2.5 group (10mL), which lowered the SPE cartridge load while maintaining comparable precision. Therefore, a powder-to-water ratio of 1:2 (2 g milk powder with 4 mL water at 45 °C) was selected as the optimal condition. This condition optimizes economic and operational efficiency while preserving extraction efficiency and analytical precision, offering reliable pretreatment parameters for high-throughput cereulide analysis in infant formula. Notably, the Austrian Agency for Health and Food Safety (AGES) has supplemented the analytical method for cereulide, recommending a solid–liquid ratio consistent with the experimental findings (1:2) [24]. This recommendation is consistent with optimized conditions and supports its practical relevance.

After optimizing the powder-to-water ratio, the extraction solvent and extraction sequence were further evaluated. Infant formula differs from many conventional food matrices because it is a processed dairy powder containing proteins, lipids, carbohydrates, fortified nutrients, and encapsulated ingredients. One relevant example is the food safety incident about Nestlé products, in which cereulide contamination was traced to arachidonic acid (ARA) ingredients added as nutritional supplements. ARA is commonly incorporated into infant formula via microencapsulation. In this process, water-soluble food-grade polymers, such as maltodextrin, whey protein, and β-cyclodextrin, are used as wall materials to form a physical barrier that encapsulates ARA and other nutrients. In conventional procedures such as ISO18465:2017, samples are commonly extracted directly by organic solvents. However, when infant formula is extracted with acetonitrile, the encapsulated components may not dissolve completely but forms a heterogeneous suspension in which milk proteins rapidly denature and aggregate into a porous micellar network together with clustered fat globules. In this aggregated structure, partially undissolved particles and protein–lipid complexes may entrap cereulide, limiting solvent accessibility and resulting in incomplete extraction. Therefore, it is necessary to change the extraction solvents, components and methods.

To compare different extraction strategies, infant formula samples were extracted using acetonitrile, 0.3% acetic acid–acetonitrile, 1% acetic acid–acetonitrile, and reconstituted solutions with deionized water at room temperature, 45 °C, and 60 °C, each followed by three times the volume of acetonitrile; previous studies have shown that protein precipitation is most effective at a water-to-acetonitrile ratio of approximately 1:3 [25]. LC-MS analysis of the positive samples indicated that neither 100% acetonitrile nor acidified acetonitrile achieved complete extraction of cereulide, with mean concentrations remaining around 1 ng·mL^−1^ (as shown in Figure 2b). Contrary to the initial hypothesis, acetic acid did not improve cereulide extraction. The stability of microcapsules and protein–fat aggregation still prevents solvent access, indicating mild acidification insufficient to release cereulide. In contrast, using initial hydration in water before acetonitrile addition resulted in a roughly fourfold increase in the target compound.

This improvement can be explained by the reconstitution behavior of infant formula during hydration. Infant formula powders are dehydrated emulsions composed mainly of proteins, fat, carbohydrates, vitamins, and minerals, and their reconstitution is strongly influenced by powder hydration and dispersion behavior [20,22]. Water addition allows the dry matrix to swell and redisperse, promoting the dissolution of lactose and whey proteins and the reorganization of casein micelles and fat globules into a milk-like colloidal system [22,23,26]. Such matrix swelling and reconstitution likely weaken the physical entrapment of cereulide within aggregates of proteins and lipids or encapsulated nutrient structures, thereby increasing solvent accessibility to the target analyte before protein precipitation. Subsequent acetonitrile addition collapses the hydrated colloidal structure and precipitates proteins, facilitating the partitioning of cereulide into the organic phase. This sequential hydration–precipitation process maximizes extraction efficiency and minimizes matrix effects.

Although experiments showed that hydration temperature (room temperature, 45 °C, and 60 °C) did not significantly affect extraction efficiency, hydration performance and operational safety were also considered. Moreover, previous studies indicate that milk powder hydration is optimal at 40–50 °C [27]. Consequently, 45 °C was chosen as the final experimental condition.

### 2.2. Optimization of the SPE Clean-Up Procedure

#### 2.2.1. Optimization of the SPE Loading Conditions

After addressing the extraction challenges associated with microencapsulation, variability in the determination results of the target analyte was still observed, indicating the need to further consider the complex matrix effects. Therefore, solid-phase extraction, a commonly used cleanup technique, was introduced. For cereulide, a hydrophobic cyclic peptide, HLB-P cartridges were selected due to their retaining hydrophobic analytes while reducing co-extracted matrix components. This approach effectively reduces matrix effects in mass spectrometric analysis and improves both sensitivity and accuracy. Accordingly, the subsequent work focused on optimizing each step of the SPE procedure to minimize matrix interference. Based on the established composition and sequence of the extraction solvent, the ratio of aqueous to organic phases remains a critical factor influencing the adsorption efficiency of cereulide on SPE cartridges. Therefore, an evaluation of cereulide adsorption on HLB-P cartridges under different water–acetonitrile ratios was conducted.

To evaluate the effect of the aqueous-to-organic ratio on SPE retention, 1 g control infant formula sample was added with 1 ng·mL^−1^ cereulide standard solution. Extractions were performed at a constant total volume using seven different aqueous phase proportions (10%, 20%, 30%, 40%, 50%, 60%, and 70%). Samples were vortexed for 5 min after being mixed with deionized water to ensure full hydration, followed by addition of the corresponding volume of acetonitrile to precipitate proteins. After 30 min vortexing and subsequent centrifugation, the supernatant was subjected to SPE. The eluates from the SPE loading step were quantified using an external standard method to assess the effect of water content on cereulide adsorption. All treatments were performed in triplicate (n = 3).

**Figure 3:**
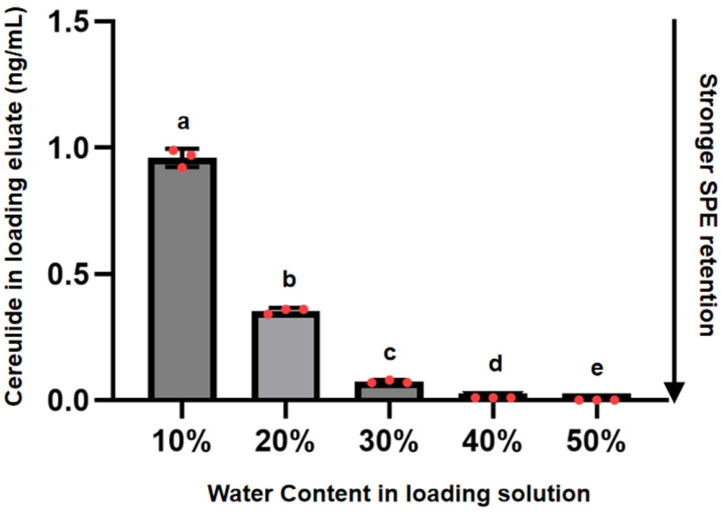
Effect of aqueous phase proportion on cereulide retention. Cereulide concentrations were measured in eluates collected from the SPE loading step; lower concentrations indicate stronger retention on the HLB-P cartridge. Data are shown as mean ± SD (n = 3). Lowercase letters (a, b, c, d, e) indicate the results of multiple comparisons after one-way ANOVA; groups labeled with different letters are significantly different (*p* < 0.01).

Quantification results showed a cereulide concentration range of 0 ng·mL^−1^–0.96 ng·mL^−1^ as shown in Figure 3. The 10% water group had the highest mean concentration (0.96 ng·mL^−1^), followed by 20% (0.35 ng·mL^−1^), 30% (0.07 ng·mL^−1^), and 40% (0.01 ng·mL^−1^), while no cereulide was detected in the 50% water group. One-way ANOVA indicated highly significant differences among the groups (*p* < 0.01), confirming that the aqueous-to-organic ratio significantly affects HLB-P cartridge adsorption efficiency. As the water content increased from 10% to 50%, the amount of cereulide detected in the loading eluate progressively decreased, suggesting enhanced adsorption of cereulide on the sorbent. This behavior is consistent with the retention mechanism of reversed-phase SPE, in which hydrophobic analytes are retained mainly through hydrophobic interactions with the sorbent. A high proportion of acetonitrile in the loading solution increases solvent elution strength and may weaken analyte–sorbent interactions, whereas an increased aqueous fraction favors the retention of hydrophobic compounds [28,29]. Notably, at a water-to-acetonitrile ratio of 1:1, cereulide was not detected in the loading eluate, indicating effective retention on the HLB-P cartridge under this loading condition. This ratio produces a clarified extract for loading while maintaining sufficient hydrophobic interaction. This outcome is consistent with the recommended operating instructions provided by the cartridge manufacturer.

Extracts containing 60% and 70% water remained visibly turbid after centrifugation and were therefore excluded from quantitative SPE loading tests, likely because of lipid emulsification and residual proteins in the infant formula matrix. These suspended matrix components may block sorbent pores or reduce mass transfer between cereulide and the active sites of the cartridge. Therefore, the limitation observed at excessive water content is more likely related to matrix-induced turbidity and sorbent fouling. Accordingly, the 60% and 70% water treatment groups were excluded from further analysis.

These results suggest that the optimal solvent composition differs between the extraction and SPE loading steps. Previous studies have shown that protein precipitation is most effective at a water-to-acetonitrile ratio of approximately 1:3, which promotes the release of protein-bound cereulide [25]. Therefore, a stepwise adjustment strategy was adopted in this study. Protein precipitation was first performed at a water-to-acetonitrile ratio of 1:3 to enhance cereulide release. After centrifugation, water was added to adjust the final water-to-acetonitrile ratio to 1:1 before SPE loading, thereby achieving optimal adsorption during purification. Under these conditions, cereulide extraction and reproducibility were satisfactory, and this protocol was selected for subsequent experiments.

#### 2.2.2. Optimization of the SPE Washing Conditions

To optimize the SPE washing conditions, the methanol proportion in the washing solution was evaluated to minimize cereulide loss. In this study, methanol–water mixtures were used as washing solutions at six volume fractions (70%, 75%, 80%, 85%, 90%, and 95% methanol) with determined eluent. The washing fractions were collected for LC-MS analyses (see Materials and Methods section for details). Therefore, the cereulide concentration detected in these fractions was used to evaluate premature analyte elution.

**Figure 4:**
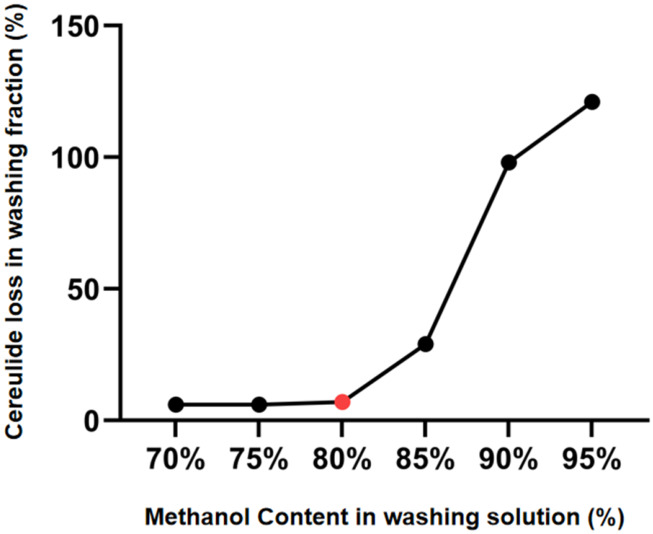
Effect of methanol content in the SPE washing solution on cereulide loss during washing. Cereulide was measured in washing fractions collected after washing with different methanol–water solutions. Lower values indicate less analyte loss during the washing step. Each value represents the mean of replicate measurements (n = 3).

The percentage of cereulide detected in the washing fractions was plotted against the methanol proportion in the washing solution to evaluate analyte loss and recovery during the SPE washing step. As shown in Figure 4, cereulide loss remained low when 70–80% methanol–water was used as the washing solution. A marked increase in cereulide loss was observed at methanol proportions above 80%, indicating that stronger washing conditions increased the risk of premature analyte elution. To maximize the washing efficiency, the critical transition point at 80% methanol was ultimately selected as the final condition.

The removal efficiency of individual lipophilic co-extractives, such as phospholipids or glycerides, was not quantitatively assessed in this experiment. Therefore, the washing condition was selected based on the behavior of the target analyte during washing and the overall validation performance of the optimized method, including recovery, precision, and matrix effects. Further non-targeted high-resolution MS analysis would be required to characterize the removal of specific lipophilic matrix components in detail.

#### 2.2.3. Optimization of the SPE Elution Conditions

To achieve efficient desorption of cereulide while minimizing matrix effects, the elution conditions of the HLB-P cartridge were optimized. A 9:1 (*v/v*) acetonitrile–methanol solution was selected as the elution solvent based on the manufacturer’s recommendation and subsequent experimental verification.

Compared with methanol alone, this solvent system provided satisfactory cereulide recovery and was therefore selected for final elution. The small proportion of methanol improved desorption efficiency by enhancing interactions with polar sites on the sorbent, whereas the acetonitrile-dominant composition helped maintain selectivity. Therefore, this composition was considered appropriate for efficient and reproducible elution of cereulide.

### 2.3. LC-MS Optimization

A preliminary study was performed to obtain the best instrumental conditions affording high resolution and short analysis time with a suitable analyte separation before testing the pretreatment efficiency. The UPLC-MS/MS method was set up for this purpose, acquiring mass spectra and adjusting columns, mobile phase and instrument parameters for commercially available standard solutions of cereulide.

**Figure 5:**
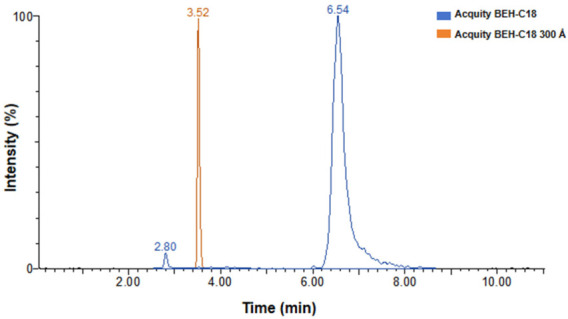
Overlay comparison of cereulide chromatographic peak profiles obtained using two columns under the same UPLC–MS/MS conditions. Different colors represent different columns.

Two reversed phase columns (Acquity BEH-C18 50 mm × 2.1 mm, dp: 1.7, Waters, Milford, MA, USA; Acquity BEH-C18 300 Å, 100 mm × 2.1 mm, dp: 1.7 μm, Waters, Milford, MA, USA) were tested to achieve chromatographic separation of the cereulide. Under these experimental conditions, the 300 Å C18 column provided a more favorable peak shape and chromatographic efficiency for cereulide (see Figure 5). This may be related to the relatively large and hydrophobic cyclic depsipeptide structure of cereulide, for which a larger pore size can facilitate analyte diffusion within the stationary phase. In addition, the Bridged Ethyl Hybrid (BEH) particle provides high mechanical stability, providing excellent column stability and reproducibility.

A high proportion of acetonitrile was used in the mobile phase to ensure efficient elution of the highly hydrophobic cereulide and to minimize the matrix effect. Formic acid improved ionization efficiency and peak shape, while ammonium acetate promoted the formation of stable ammonium adduct ions ([M + NH_4_]^+^), thereby enhancing detection sensitivity and quantitative reproducibility. Apart from the mobile phases used for the separation, the injection volume was optimized to 2 µL, because larger injection volumes increased the matrix effect.

Both 0.1 mg/L cereulide and internal standard solutions were used for parameter validation. The solutions were introduced into the mass spectrometer by flow injection to optimize parameters such as cone voltage and collision energy. According to the requirements of Commission Decision 2002/657/EC and GB/T 27404-2008 (Guidelines for Laboratory Quality Control in Physicochemical Analysis of Food in China), two ion transitions were selected for qualitative and quantitative analysis. Table 1 shows the intact list of precursor and product ions, as well as the retention time and the optimized collision energy.

### 2.4. Method Validation

#### 2.4.1. Linearity

The calibration standard solutions were analyzed under the instrumental conditions described in Section 4.4. Linear regression was performed by plotting the concentration ratio of analyte to internal standard (*x*) against the corresponding peak area ratio (*y*). The calibration curve and correlation coefficient (R^2^) for cereulide were obtained accordingly. The results showed that cereulide exhibited good linearity within the concentration range of 0.1–10 μg·L^−1^. The linear regression equation was

*y* = 0.975584*x* + 0.00576684, (1)

with a correlation coefficient R^2^ > 0.999, indicating excellent linear performance of the calibration function.

#### 2.4.2. Detection and Quantification Limits

Under the optimized conditions, the LOD and LOQ of cereulide in infant formula were 0.03 μg·kg^−1^ and 0.1 μg·kg^−1^, respectively. These results indicate that the developed LC–MS method provides sufficient sensitivity for the determination of trace levels of cereulide in complex dairy matrices. These conditions also meet the EFSA threshold for cereulide in reconstituted (liquid) infant formula (0.054 μg·L^−1^), demonstrating that the method is suitable for reliable determination.

#### 2.4.3. Matrix Effects

Matrix Effects can significantly influence quantitative accuracy and are dependent on both analyte properties and matrix composition [30]. The matrix effect was assessed by comparing peak area ratios obtained from matrix-matched standards with those from solvent-based standards at equivalent concentrations. Values within 80–120% were considered negligible, whereas values <50% or >150% indicated pronounced effects [31].

**Figure 6:**
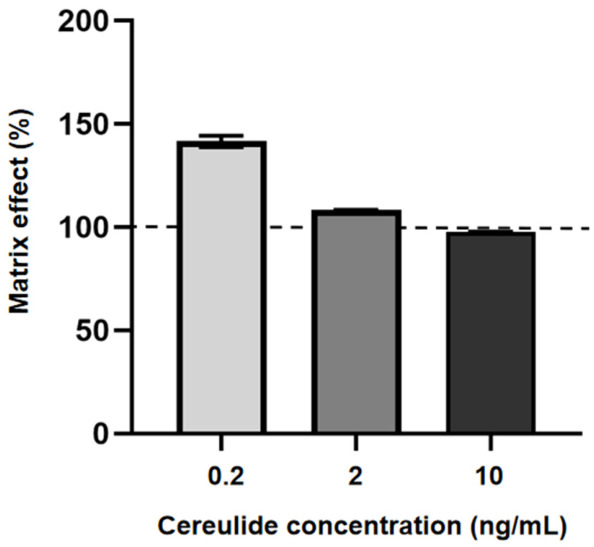
Matrix effects of cereulide in infant formula at three levels. Matrix effects were calculated by comparing the peak area ratios of matrix-matched standards with those of solvent-based standards at equivalent concentrations. The dashed line indicates 100%, representing no matrix effect. Values > 100% indicate signal enhancement, and values < 100% indicate signal suppression. Data are presented as mean ± SD (n = 3).

As shown in Figure 6, the matrix effects at low, medium, and high concentration levels were 141.5%, 108.2%, and 97.6%, respectively. The matrix effects value of 141.5% observed at the low concentration level indicates signal enhancement rather than ion suppression. In LC–ESI–MS analysis, matrix effects are mainly caused by co-eluting matrix components that alter the ionization efficiency of the target analyte [32,33]. Considering the composition of infant formula, the possible co-eluting contributors include residual lipids, phospholipids, fatty acids, glycerides, short peptides, and lipid-associated fortified ingredients such as DHA/ARA-related components [20,34,35]. Such components may influence droplet formation, solvent evaporation, surface activity, and charge competition in the ion source, leading to either signal suppression or signal enhancement depending on their physicochemical properties and elution behavior [32,33]. Therefore, the observed signal enhancement indicates that some residual matrix components from infant formula co-eluted with cereulide and promoted its ionization under the selected LC–MS conditions. The stronger signal enhancement at the low concentration level may also be related to the lower absolute analyte response, for which small changes in ionization efficiency caused by co-eluting matrix components can produce a larger relative change in peak area. In contrast, at medium and high concentration levels, the analyte response was less affected by the same level of residual matrix background.

To compensate for variability induced by matrix residues, an isotopically labeled internal standard was employed for calibration, thereby improving quantitative reliability, resulting in matrix effects values closer to 100%.

#### 2.4.4. Trueness and Precision

Method trueness and precision were evaluated using spiked infant formula samples at three concentration levels.

At 0.5 and 1.0 μg·kg^−1^, the average recoveries were 106.3% and 110.3% (shown in Table 2), with RSDs of 3.7% and 3.2%, respectively, indicating good accuracy and excellent repeatability at low and medium concentration levels. At 10.0 μg·kg^−1^, the recovery was 94.4%, with an RSD of 1.3%, indicating acceptable trueness and good repeatability at the high concentration level. Overall, recoveries ranged from 94.4% to 110.3%, and RSDs were below 3.7%.

According to commonly accepted analytical performance criteria, recoveries between 70% and 120% and RSD values below 20% are considered acceptable. In this study, all RSD values were well below 20%, confirming good precision.

### 2.5. Application

**Figure 7:**
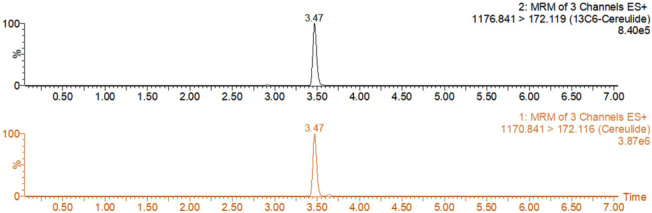
Representative MRM chromatograms of cereulide and ^13^C_6_-cereulide in a naturally incurred cereulide-positive infant formula control material. The sample was one of the control materials, with a supplier-assigned value of 0.33 μg·kg^−1^ and a measured value of 0.37 μg·kg^−1^. The chromatograms correspond to the monitored MRM transitions for cereulide (**bottom**) and the isotope-labeled internal standard (**top**) under the optimized SPE–UPLC–MS/MS conditions.

The suitability of the method was further evaluated by analyzing manufacturer-provided quality control samples using the optimized procedure (Figure 7). Two concentration levels of cereulide (0.33 μg·kg^−1^ and 2.50 μg·kg^−1^) were present in the infant formula samples, and the mean measured values were 0.37 μg·kg^−1^ and 2.56 μg·kg^−1^, respectively, both within the standard uncertainty range of ±0.07 μg·kg^−1^. Representative MRM chromatograms obtained from the cereulide-positive infant formula with an assigned value of 0.33 μg·kg^−1^ are shown in Figure 7. These results showed good agreement with the supplier-assigned values and supported the applicability of the optimized method to naturally incurred infant formula matrices.

Beyond demonstrating method applicability, these results are particularly relevant in light of recent international food safety events involving cereulide contamination in infant formula, which have led to multi-country product recalls and reported cases of infant illness. Infants represent a highly vulnerable population, and EFSA has established an acute reference dose (ARfD) of only 0.014 μg·kg^−1^ body weight, indicating that even trace-level contamination may pose a potential health concern. Moreover, risk assessments suggest that cereulide concentrations on the order of 0.05 μg/L in ready-to-consume formula may already approach levels of concern.

In this context, the ability of the proposed method to quantify cereulide at low μg·kg^−1^ levels in complex infant formula matrices provides a practical analytical tool for both routine quality control and risk-based surveillance. This is particularly important given that cereulide is heat-stable and may persist even after bacterial inactivation, making direct toxin measurement essential for ensuring product safety.

Therefore, the method not only demonstrates good analytical performance but also addresses a current gap in monitoring capability highlighted by recent contamination incidents, supporting more effective detection, risk assessment, and timely intervention in the infant formula supply chain.

## 3. Conclusions

In this study, a sensitive and reliable SPE–UPLC–MS/MS method was developed and validated for the determination of cereulide in infant formula. By integrating hydration-assisted extraction, optimized SPE purification, and improved chromatographic separation, the proposed workflow enhances the applicability of cereulide analysis to the complex infant formula matrix. The method exhibited satisfactory analytical performance in terms of linearity, sensitivity, recovery, and precision. The developed method was successfully applied to the analysis of commercial infant formula samples. The proposed method provides a useful analytical tool for the monitoring of cereulide contamination in dairy products and may contribute to improved food safety surveillance and risk assessment.

## 4. Materials and Methods

### 4.1. Chemicals and Reagents

Cereulide standard solution (C_57_H_96_N_6_O_18_, CAS No. 157232-64-9; 100 μg·mL^−1^ in acetonitrile) and ^13^C_6_-cereulide internal standard solution (C_51_^13^C_6_H_96_N_6_O_18_, CAS No. 1487375-69-8; 20 μg·mL^−1^ in acetonitrile) were purchased from Alta Scientific Co., Ltd. (Tianjin, China). For the SPE, HLB-P (hydrophilic–lipophilic balance) cartridges and WHP syringe filters (0.22 μm, 13 mm) were purchased from ANAVO Scientific Co., Ltd. (Beijing, China). For HPLC–MS/MS analysis, chromatographic separation was achieved on an ACQUITY Premier Peptide BEH C18 column (300 Å, 100 mm × 2.1 mm i.d., dp: 1.7 μm) from Waters (Waters Corporation, Milford, MA, USA). Methanol, acetonitrile, and ammonium acetate for the preparation of mobile phases were of LC-MS grade and were supplied by ANAVO Scientific Co., Ltd. (Beijing, China). Deionized water (18.2 MΩ·cm) was obtained from a Milli-Q water purification system (Merck Millipore, Burlington, MA, USA). Naturally incurred cereulide-positive infant formula quality control samples (0.33 μg·kg^−1^ and 2.50 μg·kg^−1^) were purchased from NCS testing technology Co., Ltd. (Beijing, China). Infant formula blank samples (without toxins) were purchased from a local supermarket.

Data acquisition and processing were performed using MassLynx software (version V4.1, Waters Corporation, Milford, MA, USA). Statistical analysis was performed using GraphPad Prism version 11, Microsoft Excel 2021 and Microsoft PowerPoint 2021.

### 4.2. Preparation of Standard Solutions

#### 4.2.1. Preparation of Intermediate Standard Solutions

A 0.10 mL volume of the cereulide standard stock solution (100 μg·mL^−1^) and the ^13^C_6_-cereulide internal standard stock solution (20 μg·mL^−1^) were added into separate 10.0 mL amber volumetric flasks. The solutions were diluted to volume with acetonitrile to obtain intermediate standard solutions with concentrations of 1.0 mg/L (1 μg·mL^−1^) and 0.2 mg·L^−1^ (0.2 μg·mL^−1^), respectively. The solutions were stored at −20 °C.

#### 4.2.2. Preparation of Working Standard Solutions

Prepare 1.0 mL of the cereulide intermediate standard solution (1 μg·mL^−1^) in a 10 mL amber volumetric flask and dilute to volume with acetonitrile to obtain a working standard solution of 0.1 μg·mL^−1^.

Prepare 5.0 mL of the ^13^C_6_-cereulide internal standard intermediate solution (0.2 μg·mL^−1^) in a 10 mL amber volumetric flask and dilute to volume with acetonitrile to obtain an internal standard working solution of 0.1 μg·mL^−1^.

#### 4.2.3. Preparation of Calibration Standard Solutions

Transfer 10.0 μL, 50.0 μL, 100.0 μL, 200.0 μL, 500.0 μL, and 1000.0 μL of the 0.1 μg·mL^−1^ working standard solution into separate 10.0 mL volumetric flasks. Add 200.0 μL of the 0.1 mg/L ^13^C_6_-cereulide internal standard working solution to each flask and dilute to volume with the initial mobile phase to obtain a series of calibration standard solutions with concentrations of 0.1, 0.5, 1.0, 2.0, 5.0, and 10.0 μg·L^−1^, each containing 2.0 μg·L^−1^ of internal standard.

### 4.3. Sample Preparation

#### 4.3.1. Sample Pretreatments

A 2 g sample was collected from homogeneous infant formula, and 20.0 μL of internal standard working solution (0.1 μg·mL^−1^) was added. After standing for 30 s at room temperature, 4 mL of deionized water (45 °C) was added, followed by vortex mixing (5 min). Then 12 mL of acetonitrile was added, and the mixture was shaken for 30 min. The extract was centrifuged at 12,000 r/min for 10 min at 4 °C. The entire supernatant was transferred to a 50.0 mL centrifuge tube and 8 mL of water was added to adjust the water–acetonitrile ratio to 1:1 for SPE.

#### 4.3.2. Extraction and Purification Method

The HLB-P cartridge was conditioned with 6.0 mL methanol and 6.0 mL deionized water. The entire supernatant (24 mL) obtained above was loaded onto the cartridge; after passing through, the cartridge was washed with 6.0 mL of 50% methanol in water followed by 6.0 mL of 80% methanol in water. The analytes were then eluted with 6.0 mL 9:1 (*v*/*v*) acetonitrile-to-methanol solution. All eluates were collected and evaporated to dryness under a gentle stream of nitrogen and resuspended in 1.0 mL acetonitrile, filtered through a WHP microporous membrane (0.22 μm, 13 mm) before their analysis by UPLC-MS.

### 4.4. LC-MS/MS Analysis

Chromatographic separation was performed using an ACQUITY UPLC system coupled to a Xevo TQ-S mass spectrometer (Waters Corporation, Milford, MA, USA) equipped with an electrospray ionization source operated in positive mode. Separation was achieved on an ACQUITY UPLC Peptide BEH C18 column (2.1 mm × 100 mm, 1.7 μm) maintained at 40 °C, with the autosampler temperature set at 4 °C. A binary gradient consisting of (A) acetonitrile and (B) 2 mmol·L^−1^ ammonium acetate containing 0.1% formic acid was established, and the injection volume was 2.0 μL, with a flow rate of 0.40mL·min^−1^. The elution profile was 80% A–100% A (0–4.0 min), 100% A (4.0–6.0 min), and 100% A–80% A (6.0–7.0 min), followed by equilibration to 8.0 min.

Multiple Reaction Monitoring (MRM) mode was applied; an overview is given of the specific MS parameters at the selected ionization mode (ESI+) in Table 1. The capillary voltage was optimized at 3.2 kV for ESI-positive; source temperature: 150 °C; desolvation temperature: 500 °C; desolvation gas flow rate: 1000 L/h; collision cell pressure: 0.36 Pa.

**Figure 8:**
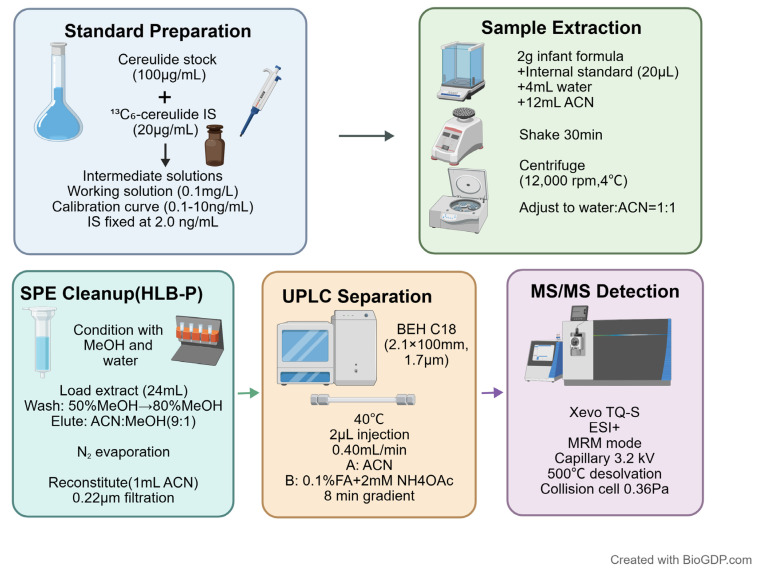
Schematic workflow of the optimized SPE–UPLC–MS/MS method for cereulide determination in infant formula [36].

Overall, the schematic illustration of the principle of the SPE–UPLC–MS/MS for the analysis of cereulide is shown in Figure 8.

### 4.5. Method Validation

For validation of the extraction and quantification method, several analytical parameters were calculated, such as linearity, recovery, limits of detection (LOD) and quantification (LOQ), trueness and precision.

#### 4.5.1. Linearity

Linearity was evaluated by preparing calibration curves (standard calibration curve) over a concentration range of 0.1–10 µg·L^−1^. Six levels were included—0.1, 0.5, 1.0, 2.0, 5.0, and 10.0 µg·L^−1^—each containing 2.0 µg·L^−1^ of internal standard. The correlation coefficient (R^2^) was used to assess the linearity of the calibration curve.

#### 4.5.2. Limits of Detection and Quantification

The limits of detection (LOD) and quantification (LOQ) were determined based on signal-to-noise (S/N) ratios of 3 and 10, respectively, under the optimized analytical conditions.

#### 4.5.3. Matrix Effects

Control infant formulas as the blank matrix from different brands were pretreated according to the procedure described in Section 4.2 to obtain matrix extracts. Afterwards, these extracts were added to prepare matrix-matched standard solutions at three concentration levels (0.2, 2.0, and 10.0 μg·L^−1^). Corresponding standard solutions at the same concentrations prepared in acetonitrile were used as solvent controls.

Matrix effects were calculated by comparing the peak area ratios obtained from matrix-matched standards with those from solvent standards at equivalent concentrations.

#### 4.5.4. Trueness and Precision

Method trueness and precision were evaluated by spiking blank infant formula samples at three concentration levels (0.5, 1.0, and 10.0 μg·kg^−1^) and processing them according to the procedure described in Section 4.2.

Trueness was expressed as recovery (%) by comparing the measured concentrations with the spiked concentrations. Precision was evaluated as the RSD of replicate measurements.

## Figures and Tables

**Table 1:** Overview of the specific MS/MS parameters for cereulide, measured in the ESI positive mode.

Analyte	Retention Time (min)	Precursor Ion (*m/z*)	Product Ion (*m/z*)	Ref Collision Energy (eV)
Cereulide	3.43	1170.841	172.1 *	66.0
			357.2	64.0
			314.2	62.0
^13^C_6_-Cereulide	3.43	1176.841	172.1 *	66.0
			315.0	66.0
			358.0	66.0

* Indicates the quantifier ion.

**Table 2:** Recovery and precision of the optimized method at different spiking levels.

Spiking Level (μg·kg^−1^)	Recovery (%)	RSD (%)
0.5	106.3	3.7
1	110.3	3.2
10	94.4	1.3

## Data Availability

The original contributions presented in this study are included in the article. Further inquiries can be directed to the corresponding authors.
